# An Examination of the Relationships between Psychological Resilience, Organizational Ostracism, and Burnout in K–12 Teachers through Structural Equation Modelling

**DOI:** 10.3390/bs13020164

**Published:** 2023-02-14

**Authors:** Hakan Polat, Turgut Karakose, Tuncay Yavuz Ozdemir, Tijen Tülübaş, Ramazan Yirci, Murat Demirkol

**Affiliations:** 1 Faculty of Education, Firat University, 23119 Elazığ, Türkiye; 2 Faculty of Education, Kutahya Dumlupınar University, 43100 Kütahya, Türkiye; 3 Faculty of Education, Sutcuimam University, 46050 Kahramanmaraş, Türkiye

**Keywords:** psychological resilience, organizational ostracism, burnout, K–12 teachers, exclusion, structural equation modelling

## Abstract

Psychological resilience, burnout, and ostracism are significant variables that may affect teachers’ performance and well-being. While psychological resilience is the ability of individuals to cope with the challenges of life/work and could support teachers in performing their profession, burnout (i.e., high levels of emotional exhaustion and desensitization) and ostracism (i.e., being ignored by others in the workplace) could lead to serious negative outcomes for both teachers and the educational system. Despite their significance, studies addressing the relationships between these variables are rare. Therefore, this study aimed to investigate the relationships between teachers’ psychological resilience, burnout, and organizational ostracism. The study used structural equation modeling (SEM) to test the hypothetical relationships between these variables. The participants were selected using a simple random sampling method among K–12 teachers working in Elazig, Turkey. The data were collected using Psychological Resilience Scale—Short Form, Organizational Ostracism Scale, and Burnout Syndrome Inventory—Short Form. Data obtained from 309 K–12 teachers were analyzed using path analysis. The findings showed that teachers’ psychological resilience was quite low, whilst they experienced high levels of burnout and organizational ostracism. The results also showed a negative relationship between their psychological resilience and organizational ostracism and burnout while determining a positive relationship between ostracism and burnout. Psychological resilience was determined to have a moderating role in the relationship between organizational ostracism and burnout. Implications were suggested for both research and practice.

## 1. Introduction

Individuals face various challenges throughout their lives, and the source of these challenges may be individual problems, economic difficulties, work demands, natural disasters, diseases, or various other environmental factors [1]. In the face of these challenges, some personality traits are considered to provide support, and psychological resilience is particularly cited in many studies as one of these personality traits. Individuals react differently to stressful situations they encounter throughout their lives. While some develop negative attitudes towards negative life events, others can develop positive attitudes and can adapt to these circumstances more easily. Psychological resilience is considered to play a significant role in people’s successful overcoming of these stressful life events [2].

Human capital is the most important resource that enhances an organization’s competitiveness and effective functioning. Since people seek social belonging not only in their private lives but also in their work lives as members of an organization, they desire to be accepted by the other members of their work group [3]. When they are not fully accepted by their work group, they are likely to have some negative experiences, such as social exclusion or ostracism. Williams [4] defines ostracism as a person’s being ignored, isolated, and pushed out of the group without any justification by another individual or group. Leary, Koch, and Hechenbleikner [5] stressed that ostracism is a serious problem since it is associated with a number of negative emotional states, such as sadness, loneliness, jealousy, guilt, shame, and social anxiety.

As teachers have a significant impact on the development and growth of children throughout their lives, they are also considered to have a crucial role in the development of societies [6]. Therefore, teaching is one of the most important yet stressful professions. Teachers’ professional success usually depends on their determination to educate individuals in the best way possible [6]. In order for teachers to show this determination, they need to be able to cope with the challenges they face, which particularly requires higher levels of psychological resilience because their psychological resilience can both protect them from and against psychological distress in the event of stressful or traumatic life events [7]. As in every organization, teachers may also experience ostracism in their schools, which may result in several negative outcomes such as loneliness, alienation, stress, anxiety, aggression, being late for work, or absenteeism in the workplace [8]. Hence, ostracism could lead to an increase in the risk of teacher burnout, as evidenced by some previous research [9]. Burnout, which is a negative emotional state that occurs as a result of long-term stress in work and family life, is also linked to several negative outcomes, just like ostracism [10].

## 2. Literature Review

### 2.1. Psychological Resilience

Resilience is broadly defined as having the flexibility, durability, and strength to endure setbacks and difficulties [11] or as an individual’s ability to cope with adversity or negative situations [12]. However, the literature offers different definitions regarding the concept of psychological resilience. For example, Luthar and Cichetti [11] defined psychological resilience as a dynamic process involving positive adjustment in the face of a severely challenging situation or a trauma. In addition, psychological resilience is defined as an ability that allows individuals to cope with difficulties, negativities, stress, and distress [13] and adapt in a positive way [14,15,16,17]. In another definition, psychological resilience is explained as a positive adaptation process in the face of difficulties that may arise in distressing situations [18]. Hunter and Warren [19], on the other hand, stated that psychological resilience is a learned process facilitated by a number of coping strategies (access to support, self-awareness, self-protection). Based on these definitions, it can be stated that resilience is generally expressed as a process or personality trait. However, the relevant literature lacks consensus on whether resilience is a personality trait, a process, or an outcome [20].

A review of the literature on psychological resilience shows that studies particularly focused on the psychological resilience of healthcare professionals [18,21,22,23,24,25,26,27,28] and educational employees [2,29,30,31,32,33,34,35,36,37] during the COVID-19 pandemic. Studies in the educational literature emphasize that resilience is an important personality trait that influences the teaching–learning process. Therefore, enforcing the psychological resilience of teachers during their training process is also crucial since teachers with weaker resilience may face several problems, such as burnout, anxiety, depression, and traumatic stress [26]. Gu and Day [31] stated that, with regard to the teaching profession, resilience is closely related to a strong sense of professionalism, self-efficacy, and motivation to teach, which are also necessary for them to be able to encourage their students to succeed. From this point of view, teachers with strong psychological resilience are likely to have a stronger commitment to their school and profession, have the capacity to cope with difficult conditions, be talented in behavior management such as sympathizing with distressed students, restraining from negative emotions while reinforcing positive ones, and have a sense of pride and satisfaction in enacting their job [38]. Day and Gu [30] stated that resilience in teachers is influenced and nurtured by the social environment in which they live and the organizational environment in which they work rather than being a personal characteristic determined by birth.

Existing studies investigated the psychological resilience of teachers in relation to several other variables such as job satisfaction [39,40], burnout [41,42,43,44,45], self-efficacy [41,43,46], job turnover [47,48], stress [41,44,45], and personal and professional relationships [49].

### 2.2. Organizational Ostracism

Organizational ostracism is defined as employees being ignored by other employees in the organization or being excluded from the group [50]. According to Wang, Lu, and Jiang [51], organizational ostracism is a negative situation that occurs as a result of the relations between employees and their managers. Robinson, O’Reilly, and Wang [52], on the other hand, state that isolation, pacification, or ignorance of employees by others is an inevitable consequence of work and social life. Scholars also argue that organizational ostracism can occur intentionally or unintentionally [52], and both the employee and the employer could have a role in its emergence [53]. In any case, organizational ostracism emerges as a process that significantly affects the human resources of the organization [54,55,56,57,58,59].

Three different types of ostracism are mentioned in the literature [50,60,61,62]: social exclusion, social rejection, and psychological exclusion. Social exclusion is a common phenomenon in organizations [63] and is defined as a non-violent form of social sanction (ignoring, avoiding, not including) against those who do not adhere to norms and expectations and threaten social stability [50,52,62]. Social rejection, as another type of ostracism, occurs when a group or individual openly expresses that they do not want to have a relationship with another group or individual because they do not have the desired characteristics or are disliked [62]. In the social rejection process, there is often no relationship or a short-term relationship between the rejected and the rejecting individual or group. It occurs by clearly stating that the rejected individual is not wanted and disliked as a group member [50,62]. Psychological exclusion, on the other hand, is the disregard of the individual by the other individual or the group, and in this type of exclusion, there is no relationship between the excluded and the excluding individual or group. Psychological exclusion can also be observed in different ways, such as avoiding eye contact or not responding verbally [52,62].

In the literature, organizational ostracism is mostly studied in relation to a sense of belonging and self-esteem [50], burnout [64], stress [65], organizational culture [66], inclusion [67,68,69,70,71], psychological health [55], and job turnover [27,72,73]. These studies emphasize that organizational ostracism has negative psychological, behavioral, and organizational consequences for employees [74]. Scott [53] stated that organizational ostracism causes some reactive behaviors of the excluded individual, such as incivility, aggression, and bullying. In addition, the desire to escape from situations where social exclusion or ostracism occurs also increases the intention of these employees to leave their job [27,72,75]. In addition, ostracism may decrease the employee’s will to contribute to their organization and reduce their commitment to work [76].

Studies also exist on ostracism in educational organizations and its influence on teachers [6,9,77]. It is considered that teachers facing ostracism could develop negative feelings and attitudes towards their profession and their school [6]. Organizational ostracism could also lead to an increase in teacher burnout [9,64]. As a result, the frequent exposure of teachers to organizational ostracism may weaken their interpersonal relations and lead to a decrease in school success [78] and may eventually cause them to leave the school [6].

### 2.3. Burnout

In the literature, the concept of burnout is associated with a negative reaction to chronic work stress, which is characterized by high levels of emotional exhaustion and desensitization and low levels of personal accomplishment at work [79]. Burnout is a persistent and negative work-related mood that develops gradually as a result of prolonged stress at work [10] or a chronic stress syndrome [80]. Burnout is a serious syndrome that may negatively affect human health by leading to the development of physical and psychosomatic dysfunction and depression [28,79]. Burnout, as a long-term response to chronic emotional and interpersonal stressors at work, is also defined in relation to other similar constructs, such as cynicism and professional inadequacy [10,81,82].

In addition to long-term exposure to chronic work stress, qualitative and quantitative work overload, role conflict and uncertainty, lack of control over one’s own work, and lack of social support are cited among the stressors that can lead to burnout [83,84]. In any case, burnout impairs both personal and social functioning and causes deterioration in the quality of work as well as physical and psychological health. Therefore, burnout can be costly not only for the employee but also for the organization [81].

Studies on burnout indicate that it is more frequently observed in employees performing people-oriented professions, such as teachers, social workers, nurses, and doctors [84,85], even though it can affect all professionals in different aspects [86]. It is particularly emphasized that the work-family conflict experienced by the teachers, which has become one of the significant and enduring problems of modern schools and the education system, could increase the risk of teacher burnout [87]. As such, several scholars [88,89] conducted studies on teacher burnout and evidenced the prevalence of burnout among teachers. Teacher burnout is often associated with individual, physiological, environmental, and psychological factors [29], as well as teachers’ perceived stress and their ability to cope with different challenges and demands [90]. On the other hand, providing teachers with resources to cope with stress throughout their formal and in-service training and enhancing their sense of personal competence and ability to manage stress can help prevent teacher burnout [87].

Numerous studies have been conducted on teacher burnout [9,32,91,92,93,94,95,96,97], and it is often investigated in relation to job stress [98,99,100,101,102], self-efficacy [103,104,105], and job commitment [106].

## 3. Materials and Methods

### 3.1. Purpose of the Study and Hypotheses

This study aimed to investigate the relationship between organizational ostracism, teachers’ psychological resilience, and burnout. Relational studies such as this one define the degree and direction of the relationship between two or more quantitative variables via calculating correlation coefficients [107,108]. The current study uses structural equation modeling (SEM), which combines different methods of analysis, such as multiple regression, path analysis, and factor analysis, and confirms the causality between various variables.

In some previous studies, it was concluded that the organizational ostracism of teachers caused an increase in their burnout levels [10]. In other studies, it has been reported that there is a negative relationship between psychological resilience and burnout [32,35,36,109,110]. However, to our best knowledge, the mediating effect of psychological resilience in the relationship between organizational ostracism and burnout is non-existent in the literature. Considering this gap, the following hypotheses were developed regarding the hypothetical relationships between these variables based on the theoretical background elaborated previously:

**Hypothesis** **1 (H1).** *There is a relationship between organizational ostracism and psychological resilience.*

**Hypothesis** **2 (H2).** *Psychological resilience has a relationship with burnout.*

**Hypothesis** **3 (H3).** *Organizational ostracism has an effect on burnout.*

**Hypothesis** **4 (H4).** *Psychological resilience has a mediating effect on the relationship between organizational ostracism and burnout.*

**Figure 1:**
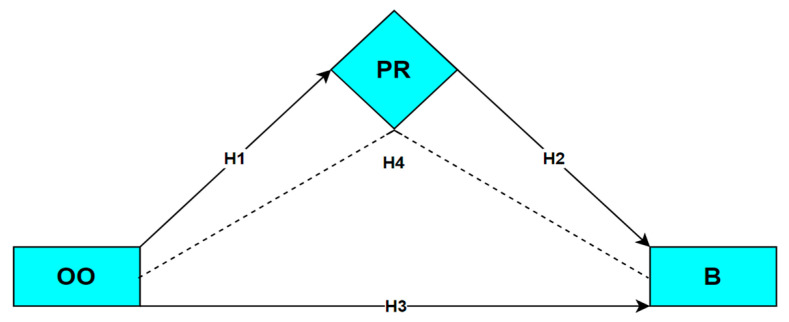
Hypothesized model of the relationships (PR = psychological resilience; OO = organizational ostracism; B = burnout).

Figure 1 illustrates the hypothetical model of the relationships between the variables investigated in the study.

### 3.2. Participants

Structural equation models are sensitive to sample size, and thus the sample size required by the structural models was taken into consideration in the current study. It is often emphasized that in such studies, the appropriate sample size should be tenfold higher than the number of items measuring the variables [111]. Accordingly, considering that the three data collection tools used in the study consisted of 30 items in total, it was agreed that the required sample size for this study should be at least 300. In order to reach a sufficient number, the scale was applied to more participants, assuming that there might be participants who filled in the research form incompletely or incorrectly. The research was carried out with the participation of 321 teachers selected by simple random sampling method among the teachers working in the city center of Elazig during the 2021–2022 academic year. During the initial review of data, 12 documents were excluded from the data set because they had extreme values, and the analysis was performed using data from 309 participants. The demographic characteristics of the participants are given in Table 1.

### 3.3. Data Collection and Data Analysis

#### 3.3.1. Measurements

Data for the current study were collected using Psychological Resilience Scale—Short Form (PRS-SF), Organizational Ostracism Scale (OOS), and Burnout Syndrome Inventory—Short Form (BSI-SF). The scales used in the study were reviewed, and their application was approved by T.C. Elazig Governorship—Provincial Directorate of National Education, with legal permission granted for the study to be performed with teachers (Permit no: 2022-63609642).

Psychological Resilience Scale—Short Form (PRS-SF):

PRS-SF was originally developed by Smith et al. [112], and its short form was developed and adapted into Turkish by Doğan [113]. The goodness of fit indices yielded by the confirmatory factor analysis (CFA) of the scale were χ^2^/df (12.86/7) = 1,83, NFI = 0.99, NNFI = 0.99, CFI = 0.99, IFI = 0.99, RFI = 0.97, GFI = 0.99, AGFI = 0.96, RMSEA = 0.05, and SRMR = 0.03. Considering these results, it can be said that the single-factor structure of the scale was preserved. In addition, the reliability coefficient of PRS-SF was previously calculated as 0.84, whilst in the current study, it was determined to be 0.97. 

Organizational Ostracism Scale (OOS):

The two-factor structure of the 14-item OOS developed by Abaslı and Özdemir [114] was tested using CFA. The fit indices of the model resulting from CFA were also tested, and the chi-square value was calculated as χ^2^ = 234.80 (df = 76, *p* = 0.00 < 0.05). Within the scope of the research, the ratio of the chi-square value to the degrees of freedom was calculated as 3.089. However, the RMSEA value was determined to be 0.067. The goodness of fit values determined for the two-factor structure of OOS was found as follows: GFI = 0.99, AGFI = 0.99, CFI = 0.99, and IFI = 0.99. These results indicate a “perfect fit” between the theoretical model and the data for the two-factor structure of the OOS. Abaslı and Özdemir [114] calculated the Alpha coefficient as 0.88 for the “Isolation” sub-dimension and 0.96 for the “Nihilation” sub-dimension. The Cronbach’s Alpha coefficient for the whole scale, including 14 items, was calculated as 0.97. In the current study, Cronbach’s Alpha coefficient was calculated as 0.94 for the “Isolation” sub-dimension, 0.97 for the Nihilation” sub-dimension, and 0.98 for the whole scale.

Burnout Syndrome Inventory—Short Form (BSI-SF):

The Turkish adaptation, validity, and reliability tests of the short version of the Burnout Scale, which was originally developed by Pines [115], were performed by Tümkaya, Çam, and Çavuşoğlu [116]. After the initial evaluation of whether the collected data allows for factor analysis (KMO = 0.91; χ^2^ = 1308.33; *p* < 0.0001), they conducted an EFA and found that the common variance values of the items ranged from 0.29 to 0.75 and only one factor with an eigenvalue greater than 1.00 emerged. The eigenvalue of this factor was 5.59 and explained 55.92% of the total variance. Ten items in the scale were loaded on the factor, with values ranging from 0.54 to 0.87. Following this process, they conducted a CFA, which yielded the same results. The reliability of the scale was tested using Cronbach’s Alpha coefficient and test-retest techniques. The internal consistency reliability coefficient of the scale was calculated as 0.91. In the present study, the internal consistency coefficient of the burnout scale was determined to be 0.97.

#### 3.3.2. Data Analysis

The data, which was collected online via Google forms, was first transferred onto SPSS 22.0 data analysis software and analyzed using AMOS 22.0 and SPSS 22.0 package programs. The maximum likelihood method was used in the AMOS package program. Sample size, multicollinearity problem, normality, and extreme values, which are the prerequisites of SEM, were first identified [117]. First of all, 19 data forms whose Z score values for the variables were not between −1 and +1 were excluded from the data set as they had extreme values. In the next step, correlation analysis was performed to determine whether there was a multicollinearity problem between the variables. If the correlation values are below 0.90, it can be said that there is no multicollinearity problem [117]. In the analysis performed, the correlations between the variables were examined, and the correlation coefficients between the variables were found to be below 0.90 (see Table 2), which indicated that there was no multicollinearity problem between the variables. In order to determine whether there is a multilinearity problem, the VIF (variance inflation factors) and tolerance values of the independent variables were also examined, and it was determined that these values did not cause multicollinearity problems (see Table 3). Another prerequisite for SEM analysis is determining whether the data exhibit a normal distribution. To test normality, the Kurtosis and Skewness values of the variables were determined, which showed that the data set exhibited a normal distribution (see Table 4).

Considering that the data showed normal distribution, the sample size was sufficient, linearity and multicollinearity problems did not exist, and the covariance matrix and the maximum likelihood methods were used in testing the measurement models and the structural model in the current study. The measurement models of the variables of psychological resilience, organizational ostracism, and burnout were tested using CFA. Whether the measurement models were validated or not was evaluated based on the Chi-square (χ^2^)/df, GFI, AGFI, CFI, RMSEA, IFI, and TLI (NNFI) fit indices. Whether the proposed hypothetical model was confirmed or not was also evaluated in detail using the specified fit indices. The fit indices were interpreted according to the value ranges presented in Table 5.

Table 5 shows that the perfect-fit index values are between 0 and 3 for χ^2^/df; ≥0.95 for GFI, AGFI, CFI, IFI, and TLI (NNFI); 0.08≤ for SRMR; and 0.05≤ for RMSEA. Acceptable fit index values are between 3 and 5 for χ^2^/df; ≥0.90 for GFI, AGFI, and CFI; 0.90–0.94 for IFI and TLI (NNFI); 0.10≤ for SRMR; and 0.08≤ for RMSEA. 

**Figure 2:**
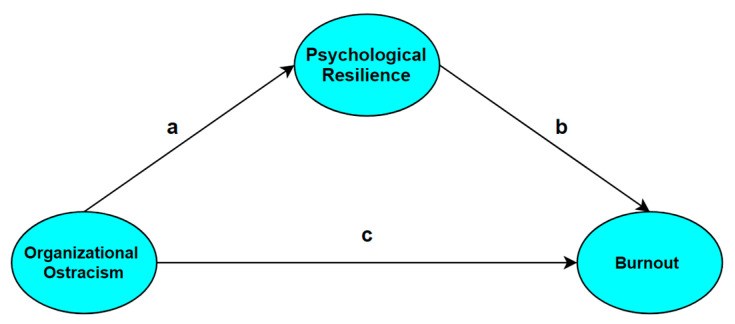
The pathways for research model.

Structural equation analysis was conducted to test the mediating role of psychological resilience in the effect of organizational ostracism on burnout. In order for psychological resilience to have a significant mediating effect on the effect of organizational ostracism on burnout;

1. The “path a” of organizational ostracism (independent variable), psychological resilience (mediating variable), and the effect of psychological resilience (mediating variable) on burnout “b way” is significant;
2. The effect of organizational ostracism (independent variable) on burnout (dependent variable) “c-way” is significant;
3. The effect of organizational ostracism (independent variable) on burnout (dependent variable) should either lose its statistical significance or there should be a significant decrease in the level of this effect [120]. The paths related to the research model are shown in Figure 2.

## 4. Results

The correlation values with regard to the scales used to collect data are presented in Table 3.

As shown in Table 3, there was a negative correlation between PRS-SF and OOS (r = −0.68, *p* < 0.01) and BSI-SF (r = −0.378, *p* < 0.01) direction. On the other hand, there was a positive correlation (r = 0.348, *p* < 0.1) between ostracism and teacher burnout levels. These results indicate that the increase in the level of psychological resilience of teachers causes a decrease in their organizational ostracism and burnout. Similarly, the increase in teachers’ organizational ostracism causes an increase in their burnout.

The VIF and tolerance values for the two variables in the measurement model are presented in Table 4.

VIF values should be less than 10, and tolerance values should be greater than 0.10 [117]. The results in Table 3 show that there is no multicollinearity problem among the variables investigated in the study. The mean, standard deviation, skewness, and kurtosis values for the variables in the measurement model are presented in Table 5.

One of the prerequisites for path analysis is that the data set exhibits a normal distribution. The analysis for the current study showed that the skewness and kurtosis values for each variable were within acceptable limits, and the data showed a normal distribution (see Table 5). The results in Table 5 show that PRS-SF values range from 1.00 to 5.00, and values have an arithmetic mean score of 1.27 (SD = 0.589). Values for the OOS range from 1.00 to 5.00, with an arithmetic mean score of 3.52 (SD = 0.699). Values on the BSI-SF scale ranged between 1.00 and 7.00, with an arithmetic mean value of 2.78 (SD = 1.289). These results indicate that teachers’ arithmetic mean scores for psychological resilience were quite low ($\overset{-}{X}$ = 1.27). On the other hand, teachers’ arithmetic mean scores for organizational ostracism and burnout are higher than their psychological resilience scores. It is extremely important that organizational ostracism and burnout scores should decrease to ensure the effectiveness of the education system. Likewise, a high level of psychological resilience is significant in enhancing teachers’ ability to cope with organizational ostracism and burnout.

### 4.1. Assessment of Measurement Model

The CFA results for the measurement tools used in SEM should be repeated with the existing data set. For this purpose, the relevant analyses of the scales used in the study were made, and the AVE (average variance extracted) and CR (composite reliability) values were presented in Table 6, whilst the fit indices were presented in Table 7.

As shown in Table 6, the Cronbach Alpha scores vary between 0.86 and 0.97. AVE values greater than 0.50 and a CR coefficient greater than 0.70 are accepted as an indication of the convergent validity and reliability of the structure [121]. Accordingly, the convergent validity and reliability of the constructs can be considered to be sufficient according to the AVE and CR values in Table 6.

PRS-SF consists of six items, and the items were accumulated in a single factor. Since the three items in the scale were reverse-coded, analysis was made by re-coding them. The measurement model of the PRS-SF was tested using first-level CFA. All of the paths related to the items in the scale were determined to be statistically significant at the 0.01 level. The fit index values were calculated as χ^2^/df = 3.4262, GFI = 0.938 AGFI = 0.914, IFI = 0.908, TLI = 0.907, CFI = 0.917, and RMSEA = 0.078. CFA results showed that the χ^2^/df, GFI, IFI, TLI, CFI, and RMSEA values were within the acceptable range of fit indices (see Table 7).

The OOS consists of 14 items and two dimensions. The first five items in the scale belonged to the dimension of “Isolation”, and the remaining nine items to the dimension of “Nihilation”. The first level CFA results showed that the paths of all 14 items in the scale were statistically significant at the 0.01 level. The fit index values of the scale were χ^2^/df = 3.694, GFI = 0.899, IFI = 0.933, CFI = 0.933, AGFI = 0.897, TLI = 0.916, and RMSEA = 0.079, which are within the acceptable range of fit indices (see Table 7).

The BSI-SF consists of one dimension with 10 items. The first level CFA results showed that all of the paths related to the 10 items in the scale were statistically significant at the 0.01 level. The fit index values for the BSI-SF were found to be χ^2^/df = 3.462, GFI = 0.899, AGFI = 0.914, IFI = 0.908, TLI = 0.907, CFI = 0.917, and RMSEA = 0.078. These results indicate that the fit index values are in the acceptable range.

### 4.2. Assessment of the Structural Model

**Figure 3:**
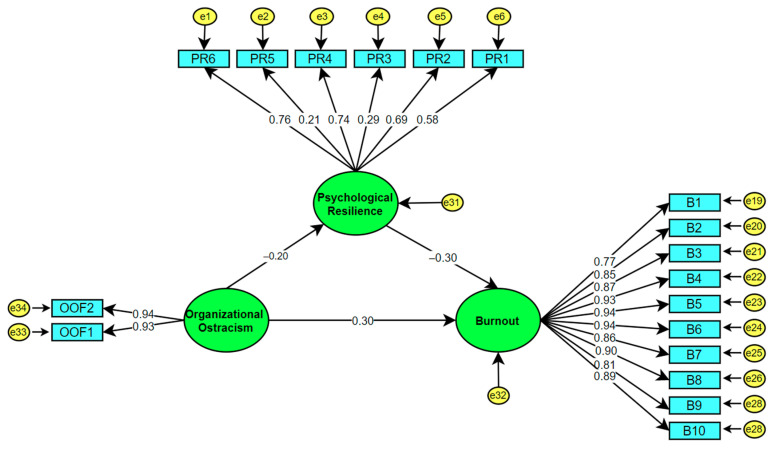
Final hypothesized model. PR = psychological resilience; OO = organizational ostracism; B = burnout; e = error variance.

The hypothetical model presented in Figure 2 was tested with structural equation modeling (SEM). Hypothetical relationships between teachers’ psychological resilience, organizational ostracism, and burnout levels were analyzed. In addition, the mediating effect of psychological resilience on the relationship between organizational ostracism and burnout was tested. The results regarding the direct and indirect relationships between the study variables were as follows: organizational ostracism had a statistically significant positive effect on burnout (t = 6.267, *p* < 0.01, β = 0.361), but a statistically significant negative effect on psychological resilience (t = −2.995, *p* < 0.01, β = −0.188). Likewise, psychological resilience had a statistically significant negative effect on burnout (t = 3.46, *p* < 0.01, β = −0.284). These results show that the hypothetical model is suitable for the mediation test. Based on these findings, the mediating role of psychological resilience in the relationship between organizational ostracism and burnout was evaluated, and the results were presented in Figure 3.

Before testing the model, some slight modifications were made in the measurement models in order to increase the fit index values of the scales. The final analysis showed that the standardized path coefficient between the BSI-SF and OOS was 0.31, between BSI-SF and PRS-SF was −0.28, and the path coefficient was −0.28. All of the paths were found to be statistically significant at the 0.01 level. 

In addition, a statistically significant positive correlation was found between organizational ostracism and burnout (see Figure 3). The fit indices of the hypothetical model were calculated as χ^2^/df = 2.901, which is in the perfect-fit range. The GFI = 0.901, AGFI = 0.899, IFI = 0.951, TLI (NNFI) = 0.941, CFI = 0.950, and RMSEA = 0.079 values were also found to be in the acceptable-fit range. These results indicated that the hypothetical model was confirmed.

Table 8 shows that the standardized regression coefficient between organizational ostracism and burnout is 0.306, which indicates a positive relationship between both variables. Organizational ostracism explains 73.2% of the total variance in burnout. According to Gignac and Szodorai [122] and Kline [111], an effect size of around 0.10 is weak, an effect size of about 0.20 is moderate, and an effect size of around 0.30 is large. In light of these specifications, findings from this study showed that organizational ostracism was a strong predictor of burnout.

The standardized regression coefficient between teachers’ organizational ostracism and psychological resilience levels was determined as −0.193 (see Table 7), which indicates that there is a negative relationship between psychological resilience and organizational ostracism. According to this finding, the increase in teachers’ psychological resilience will cause a decrease in their organizational ostracism. The psychological resilience of teachers explains the 33.8% of the variance in organizational ostracism. The standardized regression coefficient between psychological resilience and organizational ostracism shows the existence of a large effect size, which indicates that teachers’ psychological resilience significantly predicts their likelihood of experiencing organizational ostracism negatively.

In addition, while analyzing the mediating effect of resilience in the relationship between organizational ostracism and burnout, it was a prerequisite to investigate the mediating role of another variable in the relationship between the variables. As a result of the analysis, organizational ostracism was found to have a statistically significant positive effect on burnout (t = 6.267, *p* < 0.01, β = 0.361). However, organizational ostracism was found to have a statistically significant negative effect on psychological resilience (t = −2.995, *p* < 0.01; β = −0.188). Likewise, psychological resilience was found to have a statistically significant negative effect on burnout (t = 3.46, *p* < 0.01, β= −0.284). However, the results showed that the relationship between organizational ostracism and burnout decreased (β = 0.31, *p* < 0.01) with the mediating effect of psychological resilience, but this and other predictive relationships did not lose their statistical significance. This finding reveals that psychological resilience had a mediating role in the relationship between organizational ostracism and burnout.

## 5. Discussion

This study investigated the relationship between teachers’ psychological resilience, burnout, and organizational ostracism. In order to test the hypothetical relationships between these variables, structural equation modeling (SEM) was used, and the conceptual model was tested with path analysis. 

The findings of the study showed that teachers’ psychological resilience was generally low. While psychological resilience reduces the effect of negative moods on psychological health, it increases the effect of positive moods [123]. Ulukan [124], in his study with a cohort of 336 teachers, examined the relationship between happiness and resilience and found a moderately positive relationship between these variables. In the same study, it was stated that stressed and aggressive teachers exhibited lower levels of psychological resilience compared to calm and peaceful teachers. Gu and Day [31] stated that a person might show resilience in certain situations, occupations, or life stages, but the level of resilience may change in time and place. Zadok-Gurman et al. [110] investigated the effect of inquiry-based stress reduction (IBSR) intervention on teachers’ well-being, resilience, and burnout during the COVID-19 pandemic. While psychological resilience was high in the experimental group to which IBSR was applied, it was observed to be low in the control group. In a study conducted by Dereceli [125], it was concluded that stress during the COVID-19 period negatively affected psychological resilience. These results show that the psychological resilience of people was negatively affected during the COVID-19 period, most probably due to high levels of stress, fear, and boredom. COVID-19 presented a unique set of stressors and psychological trauma-related challenges for the employees. Studies investigating the effect of social support programs that would increase the psychological resilience of employees [126,127] showed that the stress experienced during the COVID-19 period negatively affected psychological resilience, so social support for the employees was crucial. Liu et al. [128] determined a high level of psychological resilience in teachers. Similarly, Gönen and Koca Ballı [129] determined a moderate level of psychological resilience in teachers. These inconsistent findings could be explained by several factors, such as the availability of social support, the nature and quality of initial teacher education, and the characteristics of school structure and climate. Moreover, as teachers in the current study exhibited a low level of psychological resilience, redesigning school structure and climate in a way to provide support and increase teachers’ resilience should be seriously regarded since resilience is a significant protective factor in the face of inevitable challenges of work life [130].

Another finding of the current study showed that teachers experienced a high level of organizational ostracism. There are studies showing that teachers are sometimes exposed to ostracism in their work context [51]. For instance, in a study on K–12 teachers by Eickholt and Goodboy [6], the effect of organizational ostracism on the commitment to school and attitudes towards the teaching profession was observed. The results of the study showed that teachers were rarely exposed to organizational ostracism, yet, when experienced, it affected teachers’ commitment to school and profession negatively. Similarly, in a study conducted by Naz et al. [64], it was determined that teachers moderately experienced organizational ostracism. When these results are considered cumulatively, it can be said that ostracism could be one of the serious problems awaiting teachers in their work environments and could result in outcomes detrimental to their well-being and performance [52,76].

The results of the current study also revealed that the levels of teachers’ burnout are high, which might not be a surprising result as they were found to have lower levels of psychological resilience and higher levels of perceived ostracism. However, these teachers could also have experienced higher levels of burnout due to some other reasons. Some studies in the literature also revealed that teachers experience high levels of burnout [94]. Burnout, defined as a response to long-term exposure to chronic emotional and interpersonal stressors at work [81], could result from their constant efforts to maintain high performance as expected from them [93]. Teachers who experience burnout are more likely to experience negative emotions in the classroom, have negative attitudes toward their students, and may feel inadequate and ineffective in coping with the problems they experience in the profession [131]. In addition, during the COVID-19 process, stress and burnout increased significantly among teachers who had to continue emergency distance education instead of face-to-face education. Vargas Rubilar and Oros [101], who examined stress and burnout with 9058 teachers during the COVID-19 pandemic period, emphasized that teachers with higher levels of stress had also experienced higher professional burnout. Moreover, they stated that factors such as lack of face-to-face contact with people, pandemic-related concerns, and excessive workload significantly increased their levels of stress, which might have caused burnout. Yu et al. [102] also found that teachers’ perceived stress was positively related to job burnout and revealed that there was a negative relationship between self-efficacy and job burnout. Their findings might indicate that teachers’ burnout could result from their lower levels of self-efficacy, and thus supporting their self-efficacy could help decrease or overcome burnout. These results support the findings of the current study and highlight the significance of devising interventions to help teachers overcome burnout to enable the sustainability of their well-being and teaching performance. Establishing a more supportive and positive school climate, building teachers’ self-efficacy and resilience through professional development incentives, eliminating stressors as much as possible, and developing teachers’ stress management capacities could help diminish their likelihood of experiencing burnout.

The current study also determined a negative relationship between organizational ostracism and psychological resilience. Yang Woon [74] stated that organizational ostracism had negative psychological, behavioral, and organizational consequences for both employees and employers. Psychological resilience, on the other hand, is defined as the individual’s positive adaptation to difficulties that arise in different aspects and phases of work [18]. While organizational ostracism is related to negative behaviors, resilience expresses a positive adjustment. Therefore, a negative relationship between these two variables could be expected. Waldeck et al. [132], in their study on resilience to ostracism, emphasized that some people may be partially supported by external factors and develop resilience to ostracism. They also identified resilience as a potential moderator in determining how people cope with ostracism. In a study conducted by Haq [133], the relationship between ostracism in the workplace, psychological capital, job performance, job stress, and intention to leave was investigated, and the results showed that there was a negative relationship between psychological capital and ostracism. Similarly, in a study by Jiang et al. [134], a negative relationship was observed between ostracism and resilience. Based on these results, it can be interpreted that teachers with lower levels of psychological resilience tend to have higher levels of perceived ostracism. Accordingly, it can be stated that helping teachers build stronger psychological resilience could also help them face the challenges in the workplace better. In fact, as the results of the current study showed, the same could hold true for burnout. The analysis yielded a significant positive relationship between organizational ostracism and burnout. When teachers are exposed to organizational ostracism, they may experience negative feelings about their work environment and the profession [6]. In their study examining interpersonal maltreatment in the workplace and burnout among teachers, Suela et al. [9] found that workplace maltreatment (ostracism, rudeness, unwanted sexual attention, abusive supervision, and undermining) was positively associated with burnout. In another study, Naz et al. [64] found a significant positive relationship between ostracism and teacher burnout.

The findings of this study exhibited that psychological resilience negatively predicts burnout, indicating that increasing the psychological resilience of teachers will decrease their burnout levels. Similar results were obtained in previous studies. For instance, in a study conducted by Fathi and Saedian [43], the relationship between teachers’ self-efficacy, resilience, and burnout was investigated, which showed that resilience negatively predicted teachers’ burnout. Likewise, Ismail et al. [32] explored the relationship between emotional, spiritual, physical, and social intelligence, resilience, and burnout. They found a negative relationship between teachers’ psychological resilience and burnout. Liu et al. [36] concluded that teachers’ resilience predicted job burnout negatively. In different studies in the literature [33,35,44,45,109,110,134], a negative relationship between teachers’ psychological resilience and their burnout was observed.

The current study also showed that psychological resilience had a mediating role in the relationship between organizational ostracism and burnout. This finding indicates that while teachers’ exposure to organizational ostracism affects their burnout positively, psychological resilience will reduce this effect and lower their burnout levels. According to the results of Jiang et al.’s [134] study explaining the relationship between ostracism and deviant behavior, as well as the mediating role of emotional exhaustion and the moderator role of resilience, resilience had a mediating effect on the relationship between ostracism and emotional burnout in the workplace. Existing studies in the literature showed that psychological resilience could be a significant moderating variable in the relationship between several other variables. For example, Uzun and Tortumlu [135] examined the mediating effect of resilience and hope in the effect of occupational burnout on life satisfaction. They determined that psychological resilience had a partial mediating effect on the relationship between burnout and life satisfaction. In the study conducted by Arpacı and Gündoğan [136], psychological resilience was found to significantly mediate the relationship between mindfulness and nomophobia. Similarly, the results of the research conducted by Liu et al. [128] revealed that psychological resilience had a mediating effect on the relationship between test anxiety and emotion regulation. These results indicate that developing teachers’ psychological resilience not only results in positive outcomes for the teachers, students, and schools but also helps eliminate the negative factors that could inhibit their success and well-being.

### Limitations and Implications

The results of the current study suggest significant implications for both teacher education and educational management professionals. As stated earlier, scholars contend that teachers’ psychological resilience could be developed during the initial teacher education and should be supported intermittently throughout their professional lives with in-service training programs. Developing teachers’ capabilities to access support, increasing their self-awareness, supporting their self-efficacy beliefs, and equipping them with resources to enable self-protection in the face of challenging events could support their well-being and performance at school [20,137]. In addition, teachers’ psychological resilience is closely associated with their social and organizational environment, and social support could leverage their resilience to a greater extent. By taking this into consideration, school administrators should strive to facilitate teachers’ resilience by constructing a more positive and supportive work environment in schools, which could also reduce the risk of exposure to negative outcomes such as burnout or ostracism.

Although the current study contributed to the literature significantly, it also bears some limitations. First, the current study was conducted in an Eastern context and in a rather centralized educational system; hence, the results could be influenced by the cultural and structural aspects of this context. However, similar studies conducted in a Western context, or comparative studies in this regard, could yield different results and enhance our understanding of their relationships and outcomes. In addition, the current study was conducted right after the COVID-19 pandemic, between the years 2021 and 2022, and the unique characteristics of the pandemic period, such as high levels of stress and low levels of social support, could have influenced participants’ behavioral and affective states. Therefore, the results of the current study could be interpreted under these circumstances, and future studies could shed light on this argument. Another limitation could have resulted from data collection via sell-assessment scales. Although participants were informed about the research purpose and about the variables tested in the study, they might have given biased answers. This, in fact, holds true for many quantitative research methods using scales to collect data but still warrants notification so that the results are interpreted accordingly.

## 6. Conclusions

The current study investigated the relationships between three variables that could have significant implications for the professional performance and psychological well-being of teachers. The teaching profession is particularly stressful in the face of increasing and fast-changing demands from modern schools and teachers’ crucial role in the development of future generations and society. As such, teachers need significant support to be able to cope with the challenges of their profession and maintain their support for the development of their students. The results of the current study are, in fact, alarming because teachers were found to experience high levels of burnout and ostracism. This indicates that teachers are not only struggling with emotional exhaustion and negative work-related mood but also lacking significant social support as they feel exposed to ostracism in their workplace. In addition, the study also indicates that teachers, unfortunately, lack the psychological resilience to cope with these challenges they face in spite of the fact that their resilience could help lower both burnout and perceived ostracism.

## Figures and Tables

**Table 1:** The demographic characteristics of the participants.

Variable	Description	f (*n* = 309)	(%)
Gender	Male	140	45.31
	Female	169	54.69
Marital status	Single	83	26.86
	Married	226	73.14
Teaching Specialty	Pre-school teaching	59	19.09
	Primary school teaching	70	22.65
	Science and math teaching	82	26.54
	Literature, arts, and humanities teaching	98	31.72
School type	Pre-school	59	19.09
	Primary school	71	22.98
	Secondary school	80	25.89
	High school	99	32.04

**Table 2:** Correlation values between the scales.

Scale	PRS-SF	OOS	BSI-SF
Psychological Resilience Scale—Short Form (PRS-SF)	1	−0.168	−0.378
Organizational Ostracism Scale (OOS)		1	0.348
Burnout Syndrome Inventory—Short Form (BSI-SF)			1

**Table 3:** VIF and tolerance values for PRS-SF and BSI-SF.

	VIF	Tolerance
Psychological Resilience Scale—Short Form (PRS-SF)	0.972	1.029
Burnout Syndrome Inventory—Short Form (BSI-SF)	0.971	1.031

VIF: variance inflation factors.

**Table 4:** Mean, standard deviation, skewness, and kurtosis values of the scales (N = 309).

Scale	Min	Max	$\overset{-}{X}$	SD	Skewness	Kurtosis
PRS-SF	1.00	5.00	1.27	0.589	1.082	1.164
OOS	1.00	5.00	3.52	0.699	0.185	−0.041
BSI-SF	1.00	7.00	2.78	1.289	0.471	−0.270

**Table 5:** Goodness of fit indices used in SEM.

Reference Sources	Fit Indices	Perfect-Fit Index Values	Acceptable-Fit Index Values
Sümer [118]; Meydan and Şeşen [119]	χ^2^/df	0 ≤ χ^2^/df ≤ 3	3 < χ^2^/df ≤ 5
Sümer [118]	* GFI	≥0.95	≥0.90
Sümer [118]	* AGFI	≥0.95	≥0.90
Meydan and Şeşen [119]	IFI	≥0.95	0.90–0.94
Meydan and Şeşen [119]	TLI (NNFI)	≥0.95	0.90–0.94
Sümer [118]	CFI	≥0.95	≥0.90
Çokluk et al. [117]	SRMR	0.08≤	0.10≤
	RMSEA	0.05≤	0.08≤

χ^2^/df: chi-square divided by the degrees of freedom; GFI: goodness of fit index; AGFI: adjusted goodness of fit index; IFI: incremental fit index; TLI: Tucker–Lewis index; NNFI: non-normed fit index; CFI: comparative fit index; SRMR: standardized root mean square residual; RMSEA: root mean square error of approximation. * Meydan and Şeşen [119] stated that the GFI and AGFI values ≥0.90 indicate a good fit, while between 0.85 and 0.89 indicate an acceptable fit.

**Table 6:** Cronbach’s Alpha, CR, and AVE scores of the scales.

Constructs		Cronbach’s Alpha	CR	AVE
Psychological Resilience Scale—Short Form (PRS-SF)		0.86	0.860	0.510
Organizational Ostracism Scale (OOS)	Isolation Dimension	0.94	0.891	0.517
	Nihilation Dimension	0.97	0.885	0.702
Burnout Syndrome Inventory—Short Form (BSI-SF)		0.97	0.975	1.328

AVE: average variance extracted; CR: composite reliability.

**Table 7:** CFA results of the scales.

Scale	χ^2^/df	GFI	AGFI	IFI	TLI	CFI	RMSEA
Psychological Resilience Scale– Short Form (PRS-SF)	3.462	0.938	0.914	0.908	0.907	0.917	0.078
Organizational Ostracism Scale (OOS)	3.694	0.899	0.897	0.933	0.916	0.933	0.079
Burnout Syndrome Inventory—Short Form (BSI-SF)	4.544	0.906	0.902	0.969	0.957	0.969	0.074

χ^2^/df: chi-square divided by the degrees of freedom; GFI: goodness of fit index; AGFI: adjusted goodness of fit index; IFI: incremental fit index; TLI: tucker–lewis index; CFI: comparative fit index; RMSEA: root mean square error of approximation.

**Table 8:** Values of variance explained, standard error, t, *p*, and standardized regression coefficients.

			Estimate	SE	t	*p*	β
BSI-SF	←	OOS	0.732	0.135	5.443	***	0.306
PRS-SF	←	OOS	−0.338	0.111	−3.040	***	−0.193

*** *p* < 0.001.

## Data Availability

The data presented in this study are available on request from the corresponding author.
